# History of development of the life-saving drug “Nusinersen” in spinal muscular atrophy

**DOI:** 10.3389/fncel.2022.942976

**Published:** 2022-08-12

**Authors:** Jiaying Qiu, Liucheng Wu, Ruobing Qu, Tao Jiang, Jialin Bai, Lei Sheng, Pengchao Feng, Junjie Sun

**Affiliations:** ^1^Key Laboratory of Neuroregeneration of Jiangsu and Ministry of Education, Co-Innovation Center of Neuroregeneration, NMPA Key Laboratory for Research and Evaluation of Tissue Engineering Technology Products, Jiangsu Clinical Medicine Center of Tissue Engineering and Nerve Injury Repair, Nantong University, Nantong, China; ^2^Department of Prenatal Screening and Diagnosis Center, Affiliated Maternity and Child Health Care Hospital of Nantong University, Nantong, China; ^3^Laboratory Animal Center, Nantong University, Nantong, China; ^4^Biomedical Polymers Laboratory, College of Chemistry, Chemical Engineering and Materials Science, Soochow University, Suzhou, China; ^5^Institute of Neuroscience, Soochow University, Suzhou, China; ^6^College of Life Sciences, Nanjing Normal University, Nanjing, China; ^7^Department of Orthopedics, The Second Affiliated Hospital of Soochow University, Suzhou, China; ^8^Nanjing Antisense Biopharmaceutical Co., Ltd, Nanjing, China

**Keywords:** spinal muscular atrophy, SMN1/2, antisense oligonucleotide, splicing regulation, nusinersen

## Abstract

Spinal muscular atrophy (SMA) is an autosomal recessive disorder with an incidence of 1/6,000–1/10,000 and is the leading fatal disease among infants. Previously, there was no effective treatment for SMA. The first effective drug, nusinersen, was approved by the US FDA in December 2016, providing hope to SMA patients worldwide. The drug was introduced in the European Union in 2017 and China in 2019 and has so far saved the lives of several patients in most parts of the world. Nusinersen are fixed sequence antisense oligonucleotides with special chemical modifications. The development of nusinersen progressed through major scientific discoveries in medicine, genetics, biology, and other disciplines, wherein several scientists have made substantial contributions. In this article, we will briefly describe the pathogenesis and therapeutic strategies of SMA, summarize the timeline of important scientific findings during the development of nusinersen in a detailed, scientific, and objective manner, and finally discuss the implications of the development of nusinersen for SMA research.

## Introduction

Spinal muscular atrophy (SMA) is an inherited neurodegenerative disease that is characterized by the progressive atrophy of the muscles innervated by the degenerated alpha motor neurons in the anterior horn of the spinal cord (Crawford and Pardo, 1996). According to recent estimates, there are about 20,000–30,000 thousand SMA patients in China, with an annual increase of 2,000 cases (Zhao S. et al., 2021). Clinically, SMA can be classified into four subtypes, I to IV, based on the time of onset and severity. Type I is the most severe, in which the patient cannot sit, develops SMA within 6 months after birth, and will die of respiratory failure before 2 years of age without medication; Type II is intermediate, with onset between 6 and 18 months of age, in which the patient can sit but cannot walk independently and shows severe complications; Type III is the mild type with a late-onset, in which the patient can walk independently; Type IV, also known as the adult-type, develops in adulthood without life-threatening complications (Farrar et al., 2013). Unfortunately, according to epidemiological surveys, type I patients account for the majority of SMA cases (Verhaart et al., 2017).

SMA has claimed the lives of countless infants and even the surviving patients need life care, which puts enormous mental pressure and financial burden on the patient’s family. The advent of Spinraza (nusinersen) represents an effective treatment approach, and it became the first drug to be approved for SMA by the U.S. Food and Drug Administration (FDA). Subsequently, two more drugs, Zolgensma^®^ (onasemnogene abeparvovec-xioi) and Evrysdi^TM^ (risdiplam) were approved by the FDA in 2019 and 2020, respectively, and multiple R&D pipelines are in the clinical stage, providing further encouragement in the search of a complete cure for SMA (Chong et al., 2021). Nusinersen is the first widely used SMA drug and is known for its excellent efficacy. Moreover, the contributions of scientists and academics to the success of nusinersen should be well known to the general public. This article will first introduce the pathogenesis and strategies for the treatment of SMA, then highlight the key scientific findings during the development of nusinersen, and finally discuss the direction of future research on the treatment of SMA.

## Pathogenesis of SMA

**Figure 1:**
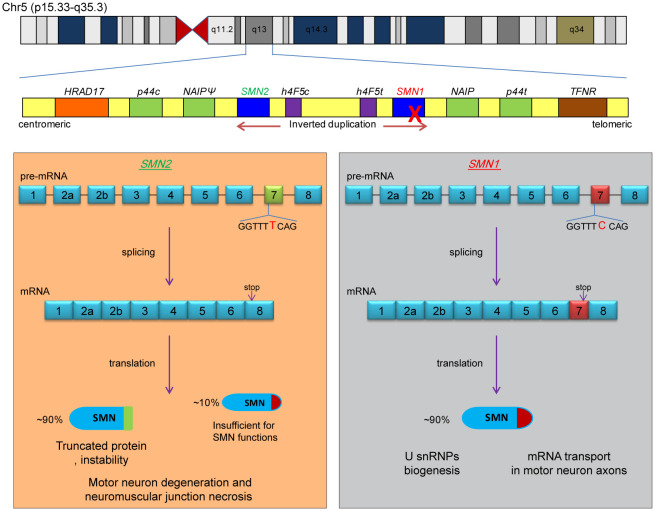
Pathogenesis of SMA. The two causative genes of SMA are located in the q13 region of human chromosome 5, which is an inverted replication region, with *SMN2* being located near the centromere and *SMN1* being located on the telomeric side (upper part of the figure). The two boxes below represent the splicing process and protein products of the *SMN1* and *SMN2* genes, respectively. The dominant nucleotide (cytosine deoxyribonucleotide of *SMN1* or thymine deoxyribonucleotide of *SMN2*) is marked in the figure, and the “stop” indicates the position of the stop codon. SMA develops because the *SMN1* gene is completely inactivated and some full-length SMN protein expressed by *SMN2* is insufficient to perform the relevant functions.

The causative gene for SMA is localized on the q13 region of chromosome 5. SMA is caused by the mutation or deletion of the *survival of motor neuron gene 1* (*SMN1*), which results in the inability to encode the survival motor neuron (SMN) protein (Lefebvre et al., 1995). SMN is a widely distributed housekeeping protein that plays a role in the assembly of the U-rich small nuclear ribonucleoprotein (U snRNP) and assists in the transport of axonal mRNAs. A recent review has comprehensively summarized the functions of SMN protein (Wirth, 2021). The 5q13 is a nearly symmetrical region that is formed by the inversion duplication of a large segment, and *SMN1* is located on one side of the telomere (Figure 1). At the centriole side of this region, there lies a parallel homolog of *SMN1*, *survival of motor neuron gene 2* (*SMN2*), which encodes only a small amount of the functional SMN protein that is not enough to compensate for the reduction of SMN caused by the deletion of *SMN1* (Lorson et al., 1999; Monani et al., 1999).

*SMN1* and *SMN2* are highly similar, with only 16 base differences being reported (Butchbach, 2021). The main difference is the sixth base of exon 7; *SMN1* has C, while *SMN2* has T. Although this single-base difference does not affect the coding of amino acids, it affects the splicing of exon 7. As a result, most of the *SMN1* gene products are full-length mRNAs containing exon 7, while the *SMN2* gene products are mRNAs with skipped exon 7 (Δ7). The stop codon of the Δ7 mRNA is displaced, and the resulting SMN is a truncated protein that is nonfunctional and highly unstable (Figure 1). Therefore, increasing the inclusion level of exon 7 of *SMN2* is one of the strategies for the treatment of SMA.

The severity of SMA depends on the dose of SMN protein. Generally, patients with mild SMA carry more copies of *SMN2* than patients with severe SMA, because a higher *SMN2* copy number means higher levels of SMN protein (McAndrew et al., 1997). In addition, other rules, such as point mutations, can affect the severity of SMA patients by altering the inclusion of exon 7. Multiple *cis*-acting splicing regulatory elements are present on the precursor mRNA of *SMN2*, which are exonic splice silencer (ESS), exonic splicing enhancer (ESE), intronic splicing silencer (ISS), and intronic splicing enhancer (ISE), regulating the inclusion of exon 7 by binding to *trans*-acting factors (Singh and Singh, 2018). SMA patients carrying the c.859G>C (p.Gly287Arg) mutation had a milder phenotype. The program ESEfinder version 3.0 predicted that the mutation created an SF2/ASF-dependent ESE (Cartegni et al., 2003; Prior et al., 2009). Subsequent experiments confirmed that this site disrupted the original hnRNP-dependent ESS, which resulted in the increased inclusion of the *SMN2* exon 7 (Vezain et al., 2010). Wu et al. (2017) reported a novel protective locus for SMA patients, the A-G transition at the –44 position on *SMN2* intron 6, which disrupted the original HuR-dependent ISS and increased the inclusion of exon 7 of *SMN2*. Moreover, altered expression of some genetic modifiers, such as plastin 3 (PLS3) and neurocalcin delta (NCALD), also possibly improves the clinical phenotype of SMA patients (Oprea et al., 2008; Riessland et al., 2017).

## Treatment Strategies for SMA

Historically, there has been a variety of treatment options for SMA, although only a few of them can be used to treat patients. Nonetheless, they are feasible and have the potential to be developed into novel drugs in the future. In the order of “Gene-RNA-Protein-Cell-Tissue,” we summarize the ideas for the development of drugs against SMA. Several excellent reviews have described some of these strategies in detail (Bowerman et al., 2017; Menduti et al., 2020; Messina and Sframeli, 2020; Chaytow et al., 2021; Iftikhar et al., 2021).

**Figure 2:**
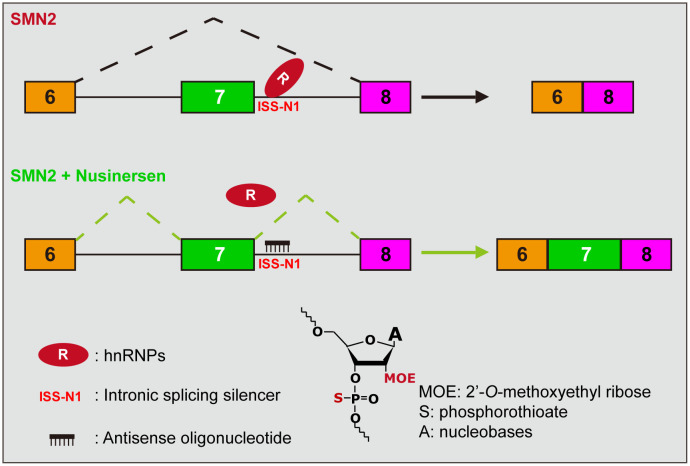
Mechanism of action of nusinersen. The presence of an ISS (called ISS-N1) near the 5’SS at the *SMN2* intron 7, combined with a repressor (hnRNP A1/2), inhibits the inclusion of exon 7, which results in the majority of splice products being SMNΔ7. Nusinersen is an antisense oligonucleotide that base-pairs with ISS-N1 to prevent the repressor binding, thereby promoting the inclusion of exon 7. The lower part of the diagram presents the chemical structure formula of ASO, where the hydroxyl group on the second carbon is replaced by MOE and an oxygen atom on the phosphate group is replaced by a sulfur atom compared to deoxyribonucleotide.

(1) Gene-Targeted Therapy: This therapy involves the delivery of complete SMN-encoding genes into cells for expression, thereby achieving the massive production of SMN protein. This protocol successfully delivered the *SMN1* gene to motor neurons in various animal models using self-complementary adeno-associated virus 9 (scAAV9) and achieved a dramatic increase in the lifespan of SMA mouse models (Foust et al., 2010; Dominguez et al., 2011). Using this strategy, the drug abeparvovec-xioi, developed by Novartis’ AveXis, was approved in the US in 2019 and entered the EU in 2020 (Mendell et al., 2021). Another solution is to increase the expression of the *SMN2* at the transcriptional level, such as using the histone deacetylase inhibitor SAHA, which has achieved the increased expression of the SMN protein in animals (Riessland et al., 2010). However, this approach has not yet been successful in clinical trials.
(2) Splicing modulation therapy: This therapeutic strategy aims to increase the functional SMN protein by increasing the inclusion level of *SMN2* exon 7. There are two approved drugs on the market, the first of which is nusinersen and the more recent one is risdiplam. The active ingredient of nusinersen is an antisense oligonucleotide (ASO), consisting of 18 2’-*O*-methoxyethyl-modified (MOE) nucleotides with phosphorothioate backbone chimerically (Figure 2). Nusinersen forms Watson-Crick base pairing with the nucleotides 10–27 of intron 7 of the *SMN2*, creating a spatial occupancy effect that prevents the binding of the splicing repressor hnRNP A1/2, thereby increasing the inclusion of exon 7 (Hua et al., 2008; Figure 2). Nusinersen binding can also unwind an occurring RNA secondary structure, which contributes to the recognition of splice sites by U1 snRNP and increases exon 7 inclusion (Singh et al., 2011, 2013, Singh et al., 2015). Risdiplam was screened against a library of millions of compounds and is an orally administered small molecule. It enhances the spliceosome recognition of exon 7 by stabilizing the ribonucleoprotein complex (RNP; Naryshkin et al., 2014; Palacino et al., 2015; Sivaramakrishnan et al., 2017). Ratni et al. (2018) have thoroughly described the chemical composition and mechanism of action of risdiplam, which will not be discussed here.
(3) Protein stabilization therapy: Cellular proteins are in a dynamic balance of synthesis and degradation. This strategy maintains the protein level of SMN by inhibiting protein degradation or inducing the read-through of stop codons. The representative drugs of this therapy are indoprofen and aminoglycosides. Some small-molecule compounds have similar functions and can greatly increase the SMN protein expression in cells, although they only slightly increase the lifespan of SMA animals (Lunn et al., 2004; Wolstencroft et al., 2005; Cherry et al., 2013).
(4) Cell replacement: This strategy aims to replenish normal neurons and restore the innervation of muscles. One option is to induce embryonic stem cells or induced pluripotent stem cells (iPSCs) into neurons or neural stem cells *in vitro* for cell transplantation (Corti et al., 2008). Another option is to induce the fibroblasts from SMA patients into iPSCs, and after genetic correction *in vitro*, to induce the iPSCs into motor neurons for autologous transplantation (Corti et al., 2012). This strategy applies to almost all neurological injury diseases, but the existing technology has not developed enough for SMA treatment.
(5) Neuroprotective therapy: This strategy aims to nourish and protect neurons. The representative drug for this therapy is riluzole. Riluzole can promote neuronal survival and axonal growth by limiting glutamate toxicity and enhancing the expression of neurotrophic factors and has been approved for the treatment of another neurodegenerative disease called amyotrophic lateral sclerosis (ALS; Katoh-Semba et al., 2002; Calabrese et al., 2021). However, riluzole insignificantly improved the median survival in SMA mice and had no protective effect on the proximal axonal loss (Haddad et al., 2003). Although a clinical trial with a small cohort showed that riluzole may benefit children with SMA, no follow-up trial results have been published (Russman et al., 2003; Chaytow et al., 2021). Overall, this is an untargeted therapeutic strategy that cannot improve SMN protein levels from the source and treat the disease.

## History of Nusinersen Development

**Figure 3:**
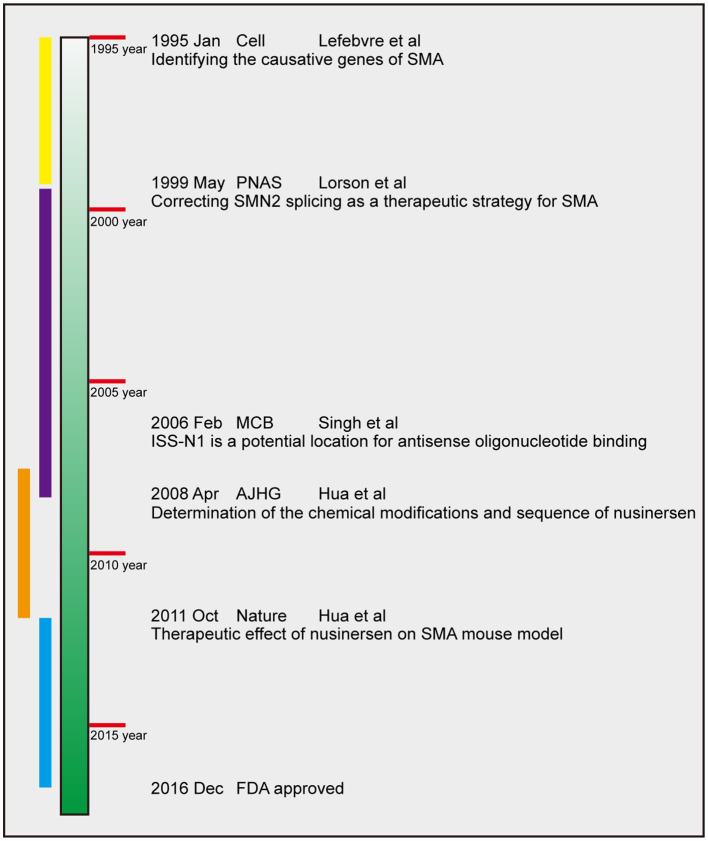
Timeline of nusinersen development. The four periods are chronologically labeled in yellow, purple, orange, and blue. The dates of publication, journals, first author and main finding of several landmark efforts are presented. PNAS, *Proceedings of the National Academy of Sciences of the United States of America*, MCB, *Molecular and Cellular Biology*; AJHG, *American Journal of Human Genetics*.

Nusinersen essentially targets the *SMN2*. Therefore, we trace the history of its development back to the discovery of the causative gene of SMA. From 1995, when the gene encoding SMN was identified, until the approval of nusinersen by the FDA in 2016, the process of research and development has spanned for over 20 years. According to the historical facts of several major discoveries and the development rule of scientific research, we divide the process of development of nusinersen into four periods, namely, gene period, splicing regulation period, animal treatment period, and clinical treatment period (Figure 3). This chapter describes the important research results during each of these four periods.

### Gene period

In the 1990s, limited by molecular cloning and DNA sequencing technology, the scientific community only knew that SMA patients carried large-scale deletions in the 5q13 region of chromosome 5 and could not determine the specific causative gene. In 1995, the French scientist Suzie Lefebvre and colleagues reported that the 5q13 region was an inverted replication segment and the region near the telomere contained a gene called *survival motor neuron* (*SMN*), which encoded a new 294 amino acid-containing protein that determined SMA (Lefebvre et al., 1995). This study provided a clear research target for this lethal disease and marked the formal entry of SMA treatment into the gene period. Subsequently, the team continued to publish articles confirming the deficiency of SMN protein in SMA patients, as well as their localization in a novel nuclear structure and interaction with RNA-binding proteins (Lefebvre et al., 1997). During this time, the structure of the SMN protein and some of its now-known biological functions were elucidated (Lorson et al., 1998; Pellizzoni et al., 1998).

By 1998, scientists had determined that two genes could encode SMN protein, namely *SMN1* and *SMN2*, and investigated the differences in sequence and splicing (in articles during this period, the names of these two genes are not uniform; SMN^C^ and SMNtel refer to *SMN1*, while SMN^T^ and SMNcen refer to *SMN2*). However, the reason for the differences in splicing patterns of the two was unclear, and no scientist considered the *SMN2* gene as a therapeutic target for SMA. Elliot J. Androphy and his postdoc Christian L. Lorson at the Tufts University’s School of Medicine were the first to propose that the products encoded by the *SMN1* and *SMN2* were not functionally identical. The SMN encoded by *SMN1* had a structural domain that was required for the aggregation of SMN itself (subsequent studies have revealed that the structural domain is the YG box, the site where SMN binds to Gemin2 and is required for the formation of spliceosomes), whereas the SMN encoded by *SMN2* could not undergo self-aggregation (Lorson et al., 1998). In a subsequent collaboration with Brunhilde Wirth, they systematically evaluated several differential bases in the *SMN1* and *SMN2*, and observed that the splicing difference in *SMN1/2* was determined by the C-to-T transition of the sixth nucleotide on exon 7. From this they deduced a clear mechanism for SMA pathogenesis, namely, that *SMN2* cannot fully compensate for the reduction of SMN protein caused by *SMN1* disruption due to the C-T transition, which leads to SMA. More importantly, they highlighted that prevention of the skipping of exon 7 could be one of the strategies for the treatment of SMA (Lorson et al., 1999). This study was the first to suggest that the activation of the *SMN2* gene had potential therapeutic application against SMA, which provided the molecular basis for subsequent drug development. Since then, the research entered the period “splicing regulation of *SMN2*”. Another article also suggested that the C-to-T substitution could affect the splicing pattern of the two genes, resulting in truncation of the protein product of *SMN2* (Monani et al., 1999). This article was published soon after Lorson’s research, with both Androphy and Lorson as the co-authors.

During that period, multiple mouse models of SMA were generated based on the elucidated pathogenesis. Li Hung lab knocked out *Smn1* in mice and knocked in two and four copies of *hSMN2*, to successfully construct severe (*Smn1*^−/−^, *SMN2*^2TG/0^) and mild (*Smn1*^−/−^, *SMN2*^2TG/2TG^) SMA mouse models. The severe SMA mice had a lifespan of approximately 10 days, showed motor deficits, and had pathological changes like those of type I SMA patients. Mice with mild SMA had a normal lifespan and motility but showed necrosis of distal tissue and were used to mimic adult SMA patients (Hsieh-Li et al., 2000). These mice were called “Taiwanese mice” and were widely used. Almost simultaneously, Burghes’ lab reported another SMA mouse model carrying two copies of *hSMN2* (*Smn1*^−/−^, *SMN2*^TG/TG^) with a lifespan of about 5 days (Monani et al., 2000). The Burghes’ team subsequently inserted the SMNΔ7 coding sequence into severe Taiwanese mice and constructed a “Δ7 SMA model” with a genetic background of (*Smn1*^−/−^, *SMN2*^2TG/0^, *SMNΔ7*^TG/TG^), which survived for about 2 weeks. These mouse models provided the basis for *in vivo* pharmacokinetic studies of late-stage SMA drugs (Le et al., 2005).

### Splicing regulatory period

The main issue faced during this period was how to correct the inclusion of *SMN2* exon 7 and use this strategy to treat SMA. From small molecule compounds to ASOs, scientists have made various attempts to do this. Although most of the studies were terminated, they still accumulated valuable experience for subsequent drug development. In fact, any major discovery is a step forward from its predecessors, which was also obvious in the case of nusinersen.

In 2001, Li Hung’s lab discovered the small molecule sodium butyrate that could promote the inclusion of *SMN2* exon 7 and reported the improvement of clinical symptoms and prolongation of lifespan in SMA mice (Chang et al., 2001). Several laboratories have screened many small molecule compounds. Among them, Jianhua Zhou in collaboration with Elliott Androphy designed a minigene labeled with GFP or luciferase to convert the inclusion/skipped of exon 7 into reporter signals by controlling the open reading frame (Zhang et al., 2001). This was the earliest *SMN2* splicing-dependent high-throughput drug screening system, and effective small molecule compounds were successfully screened. This idea of high-throughput screening using reporters was an important guide in the development of the *SMN2* splicing regulator (including nusinersen and then risdiplam). However, the early small molecule drugs were unsuccessful in clinical trials.

Another important strategy in the same period was the use of antisense oligonucleotide technology to correct the splicing of *SMN2*. Lim and Hertel used ASO to bind the 3’-splice site (SS) of intron 7 to regulate *SMN2* splicing in favor of exon 7 inclusion, based on the principle that ASO inhibited the recognition of the 3’SS of intron 7 and competitively enhanced the recognition of the 3’SS of intron 6 (Lim and Hertel, 2001). Kazunori Imaizumi’s lab reported the presence of an ISS called element 1 in the intron 6 of *SMN2*, and that ASO bound to element 1 increased the inclusion of exon 7 by a small amount (Miyajima et al., 2002; Miyaso et al., 2003). Adrian R. Krainer’s lab at the Cold Spring Harbor Laboratory (CSHL) designed small chimeric effectors [also known as peptide nucleic acids (PNAs)], a strategy that coupled nucleic acids and peptides to enhance the targeting of *SMN2* through a complementary ASO and enhance the regulation of splicing through a minimized splicing-activation domain of the SR protein, achieving efficient inclusion of exon 7 of the *SMN2* gene in cells (Cartegni and Krainer, 2003). The laboratory of Francesco Muntoni at the Imperial College of London employed a bifunctional ASO that consisted of an antisense portion complementary to the target exon and a tail that recruited splicing activators, which enhanced the inclusion of *SMN2* exon 7. Bifunctional ASO significantly increased the levels of SMN protein and numbers of Gem vesicles in cells of SMA patients (Skordis et al., 2003). However, most of these ASOs had no animal test results reported, bifunctional ASO only extended the lifespan of SMA mice by one day (Baughan et al., 2009).

A study led by Ravindra N. Singh of the University of Massachusetts Medical School was crucial to the development of nusinersen. The team identified an ISS, called the ISS-N1, at the 5’ end of intron 7 of *SMN2*, which strongly inhibited the inclusion of exon 7, and designed an ASO for this position that could correct the splicing of *SMN2* exon 7 (Singh et al., 2006). In that study, the binding position of the ASO was 7–25 of intron 7, which was different to the position 10–27 of nusinersen, and the chemical modification used was 2’-*O*-methyl (OME), which is also different to nusinersen’s MOE. Subsequent studies demonstrated that the OME-modified ASO only slightly increased the level of full-length SMN protein in the Δ7 SMA model (Williams et al., 2009). However, this ASO was ineffective in correcting *SMN2* splicing in the mild Taiwanese SMA model and caused an inflammatory response (Hua et al., 2010).

The early exploration of these ASOs failed to yield the expected results due to: (1) the lack of systematic studies to determine the optimal ASO binding site; (2) the lack of clarity as to which chemical modifications were most readily druggable.

In 2004, Dr. Yimin Hua joined the AR Krainer Laboratory of CSHL. He used the walking method (set multiple short ASOs, overlapping between adjacent ASOs) to screen ASOs of different lengths in a large-scale and systematic manner, covering the entire region from intron 6 to exon 8 of *SMN2*, and collaborated with ISIS Pharmaceuticals in California (renamed IONIS in 2015) to analyze the efficacy of various ASO chemical modifications. After screening, they finally reported that ASO 10–27 (i.e., nusinersen) as well as its extended version had the strongest antisense activity after the monomer was modified with a methoxyethyl group at the 2-position of the glycosyl group, and could correct the splicing defect of *SMN2* at lower concentrations (Hua et al., 2007, 2008; Figure 2). ASO 10–27 targeted the above-mentioned ISS-N1. Dr. Hua and Prof. Krianer further elucidated the mechanism of action of ASO 10–27, describing that there were two binding sites for the splicing inhibitor hnRNP A1/2 at this position and that ASO prevented the binding of hnRNP A1/2 through a space-occupying effect (Figure 2; Hua et al., 2008). This article marked the official establishment of the main component ASO 10–27 of nusinersen, and SMA entered the preclinical development period.

### Animal treatment period

During this phase of the development of nusinersen, the theme essentially shifted from “discovery” to “verification”. Testing the efficacy and toxicity of ASO 10–27 in mammalian and primate models was vital in determining whether the drug could be taken further into clinical trials. In 2009, Dr. Hua used the mild SMA models of Taiwanese mice to construct a severe SMA model that was more suitable for drug screening (50% SMA mice per litter, with an average lifespan of 10 days) and developed a monoclonal antibody that could specifically recognize human SMN protein which laid the foundation for animal experiments (Hua et al., 2010, 2011). ISIS has accumulated several research foundations in the biodistribution, pharmacokinetics, and toxicology of ASO. They observed that MOE-modified ASO could be distributed throughout the body with the blood circulation and was well absorbed in peripheral tissues such as the liver, which also confirmed the biological safety of ASO (Butler et al., 2000; Geary et al., 2001, 2003).

Yimin Hua et al. initially tested ASO 10–27 in healthy adult *hSMN2* transgenic mice by tail vein injection. Significant increases in the inclusion of *SMN2* exon 7 were observed in peripheral tissues such as the liver and kidney but not the central nervous system (CNS) due to the blood-brain barrier (BBB; Hua et al., 2008). This research provided initial evidence that MOE-modified ASO 10–27 could effectively correct the splicing of *SMN2* in animal tissues, although it required intracerebroventricular (ICV) or intrathecal (IT) injection to deliver ASO to the CNS. They subsequently tested the efficacy of AS0 10–27 in the CNS in mild Taiwanese SMA mice. Upon injection into the ICV, ASO 10–27 nearly reversed the splicing of *SMN2* in the brain and spinal cord and significantly alleviated pathological features such as tail docking and distal tissue necrosis (Hua et al., 2010). Importantly, the effect of ASO persisted for over 6 months after a 1-week ICV infusion at a dose of 50 μg/day (Hua et al., 2010; Rigo et al., 2014). In severe Taiwanese SMA mice and Δ7 SMA mice, ICV injection of ASO 10–27 only less than doubled the lifespan of the mice, although it substantially increased the expression of SMN and numbers of motor neurons in the spinal cord (Hua et al., 2011; Passini et al., 2011). However, merely improving the phenotype but not significantly rescuing the lifespan in SMA mice was not enough to push ASO 10–27 into clinical trials. Hua and colleagues explored the effect of different methods of administration on the therapeutic potential of ASO 10–27. Two subcutaneous (SC) ASO injections at 1 and 3 days postnatally increased the median lifespan of severe SMA mice from 10 to 250 days, while almost completely restoring the capacity of diseased mice to exercise (Hua et al., 2011). In neonatal mice, the BBB is not fully developed, and SC injection results in the systemic delivery of ASO. This study not only demonstrated the exciting therapeutic effect of ASO 10–27 but also suggested that the rescue of SMN expression in peripheral tissues was necessary for the treatment of SMA, opening a new avenue in academic research. Non-human primates lack *SMN2* and cannot be tested for target engagement of ASO by splicing tests or protein expression. High doses of ASO 10–27 (1–7 mg) were injected into the CNS of adult or juvenile cynomolgus monkeys by intrathecal injection. It was observed that ASO was distributed in all regions of the spinal cord without causing death or other adverse effects (Passini et al., 2011). These data demonstrate that ASO 10–27 already possesses essential qualities for a drug to treat SMA, establishing the basis for the entry of nusinersen into clinical trials. In 2012, Frank Rigo and Frank Bennett of ISIS and A.R. Krainer and Hua Yimin of CSHL jointly issued a article summarizing their research on ASO 10–27 between 2004 and 2012 (Rigo et al., 2012).

### Clinical treatment period

ASO 10–27 was renamed ISIS-SMNRx. Three long-term clinical trials were initiated in 2011 and were conducted in multiple hospitals, with support from ISIS Pharmaceuticals Inc. and Biogen. The results of the trials were published in 2016 and 2017, respectively.

Based on a detailed comparison of ICV bolus and IT bolus in rodents and primates, a single dose of the latter was selected for clinical trials. In the phase-1 clinical trial, the primary study was on the half-life and safety of nusinersen in 28 medically stable patients aged 2–14 years with type 2 and type 3 SMA. The participants received a single dose of 1–9 mg nusinersen as an IT injection. Nusinersen showed similar results as the preclinical trials: good tolerance, wide distribution throughout the cerebrospinal, and a half-life of >6 months (Chiriboga et al., 2016). The phase-II clinical trial used multiple doses to test the tolerability, pharmacokinetics, and clinical efficacy of higher doses (6 mg and 12 mg) of nusinersen in 20 patients with infantile SMA. The results indicated that although the higher doses resulted in multiple adverse events, they still achieved significant improvements in developmental milestones, muscle function, and motor test scores. Importantly, the autopsy results in this clinical trial revealed that nusinersen was not only widely distributed throughout the spinal cord and brain but also increased the inclusion of *SMN2* exon 7 and SMN protein levels, which is the first direct pharmacological evidence of ASO in the human CNS (Finkel et al., 2016). The Phase-III clinical trial used a randomized, double-blind, sham-controlled approach to evaluate the safety and clinical efficacy of nusinersen in a larger number of younger patients with SMA. Consistent with the first two trials, the treated children were more likely to survive and have improved motor function compared to the control group. The unique finding was that nusinersen yielded a better curative effect when administered in younger children (Finkel et al., 2017).

Nusinersen has a broader spectrum of patients. The dosing regimen of nusinersen is a 12 mg dose equivalent given as four intrathecal injections for the first 2 months, followed by refills every 4 months (Keinath et al., 2021). Infants with both type I or type II SMA showed significant improvement in exercise capacity after drug treatment that persisted for 2 years (Pane et al., 2018, 2019; Aragon-Gawinska et al., 2020). A potential concern is that a lumbar puncture could lead to complications such as hydrocephalus (Ramdas and Servais, 2020). Notably, pre-symptomatic treatment is far more effective than symptomatic (Gidaro and Servais, 2019; Servais et al., 2021). Therefore, enhanced newborn screening as well as genetic diagnosis is essential for SMA treatment at this stage. The cost of three courses of nusinersen treatment is about $2 million, which is prohibitive for many SMA patients. Fortunately, more and more countries are including it in their health insurance, which will greatly increase the beneficiary group of increased nusinersen.

## Discussion

The advent of any drug is not achieved overnight; it is a gradual breakthrough based on several previous studies. Not only for nusinersen, the multitude of findings or ideas described above also contributed to the development of abeparvovec-xioi and risdiplam. Although the availability of the drugs is a big step towards curing SMA, there are still several issues that need further investigation. Moreover, emerging approaches may introduce novel strategies for the treatment of SMA. These issues have been discussed in more detail below.

### How does depletion of SMN lead to the degeneration of motor neurons?

The downstream molecules of SMN have long been a mystery. Identifying the molecular mechanisms downstream of SMN and intervening in combination with nusinersen may help improve nusinersen efficacy and even pioneer new SMA treatment strategies. It is now understood that the most important function of SMN is to participate in the assembly of U snRNP, which is an important component of the spliceosome. Therefore, theoretically, the depletion of SMN will cause a large number of splicing abnormalities, as has been demonstrated by studies on animal models and in samples from SMA patients (Zhang et al., 2013; Huo et al., 2014; Ng et al., 2015). Several studies have highlighted the effect of SMN on the minor spliceosome, with some U12-dependent introns being abnormally spliced in SMA patient cells and animal models (Boulisfane et al., 2011; Lotti et al., 2012; Doktor et al., 2017). Follow-up studies have confirmed that virus-mediated delivery of minor snRNA genes moderately ameliorated the disease in SMA mice by reversing defects in the splicing of U12 (Osman et al., 2020). However, no downstream molecules of SMN causing the degeneration of motor neurons due to U2- or U12-splicing errors have been identified so far.

Another hypothesis supports that SMN does not have a single clear target and that the degeneration of motor neurons is the ultimate effect caused by the accumulation of numerous adverse events. Increasing evidence confirms that the reduction of SMN leads to the intracellular production of R-loop, a DNA-RNA hybrid formed when the pre-mRNAs formed by gene transcription are difficult to be separated from the template (Kim and Wang, 2021). Jangi et al. (2017) reported a substantial increase in the retention of U12-type introns, which are substrates of R-loops, in the spinal cord of SMA mice. Zhao et al. (2016) demonstrated that SMN recruits the helicase senataxin to RNA polymerase II by interacting with it, helping resolve R-loops in the transcription termination region. Kannan et al. (2018) reported that chronic low levels of SMN can cause senataxin deficiency, resulting in increased R-loops and DNA double-stranded breaks (DSBs), whereas the restoration of SMN expression improved senataxin expression and DSB accumulation in the neurons of SMA mice and SMA patient cells. A recent study reported that the overexpression of zinc finger protein ZPR1 increased the levels of senataxin, reduced the accumulation of R-loop, and rescued motor neurons in SMA mice as well as DNA damage in patient cells (Kannan et al., 2020). The explanation for the R-loop is that the low basal level of SMN leads to a reduction in the number of spliceosomes, slowing or stalling the process of splicing. Also, a large number of transcripts cannot be separated from the template to form an R-loop, which causes the accumulation of the p53 pathway and DNA damage response in SMA cells. Moreover, the depletion of SMN has been demonstrated to affect intracellular translation. The study by Fabio Lauria et al. reported that the loss of SMN leads to the depletion of ribosomes and affects the translation of some proteins, such as acetylcholinesterase, leading to motor neuron lesions (Lauria et al., 2020).

The team from Columbia University has proposed that excessive accumulation of the apoptosis-related protein P53 is among the causes of motor neuron death. In the SMNΔ7 mouse model, they observed that the phosphorylation of serine 18 of p53 selectively labeled the degenerated motor neurons and was required for the death of motor neurons, and that P53 phosphorylation was due to Stasimon deficiency to activate p38 mitogen-activated protein kinase (MAPK; Simon et al., 2017, 2019). They confirmed that deficiency of SMN caused the aberrant splicing of *Mdm2* and *Mdm4*, and exon-skipping of *Mdm2* and *Mdm4* was necessary to trigger strong p53 activation (Van Alstyne et al., 2018). In a follow-up study, they used three phenotypically distinct SMA mouse models and again confirmed that SMN-dependent missplicing of *Mdm2* and *Mdm4*, causing p53 nuclear accumulation, is a conserved pathological mechanism, while the inhibition of p53 prevents motor neuron death in severe and moderate SMA mouse models (Buettner et al., 2021). However, *Mdm2/4*-targeted therapy has unfortunately shown limited improvement in increasing the lifespan of SMA mice (Simon et al., 2017). Hence, *Mdm2* and *Mdm4* cannot be used as downstream targets for SMN yet.

It is puzzling why the deficiency of SMN causes the degeneration of motor neurons rather than interneurons or other neurons. The spinal cord is a tissue with extensive heterogeneity and there are complex interactions between glial cells and neurons, which are related to SMA (Abati et al., 2020). Assessing the sensitivity of SMN to different cell types at the cellular level is necessary, although no relevant findings have been presented due to technical limitations. Single-cell RNA sequencing (scRNA-seq) has emerged recently to analyze the transcriptional and alternative splicing changes in single cells and is extremely well suited to address cellular heterogeneity. This technology has led to revolutionary advances in cellular taxonomy and has uncovered new disease-specific cell types in several neurodegenerative diseases (Zhou et al., 2020; Sun et al., 2021). However, scRNA-seq is yet to progress in SMA, although it may lead to discoveries about the downstream molecules of SMN or degeneration of SMN-specific motor neurons in the future.

### How does the peripheral tissue affect motor neuron lesions?

Yimin Hua et al initially reported that SC injection (systemic administration) of ASO 10–27 was more effective than ICV injection (CNS administration) in extending the life span of SMA mice (Hua et al., 2011). This study led scientists to consider the importance of peripheral tissue lesions in SMA patients and mouse models. In a follow-up study, they designed an experiment, which once again confirmed that SMA was not a cell-autonomous defect of motor neurons. They systemically administered ASO 10–27 and simultaneously injected a decoy with complete base-pairing with ASO into the CNS to block the former, which restricted ASO 10–27 to only peripheral tissues, which still resulted in the extension of life span and restoration of motility in SMA mice (Hua et al., 2015). This article suggested that the supplementation of SMN protein in the CNS alone is necessary but not sufficient to treat SMA. In other words, peripheral tissues also contribute to the pathogenesis of SMA. To date, numerous studies have focused on the peripheral tissue lesions of SMA in patients and animal models (Yeo and Darras, 2020). Pathological changes and molecular mechanisms in most tissues such as peripheral nerves (Hunter et al., 2014), heart (Sheng et al., 2018), liver (Szunyogova et al., 2016; Wan et al., 2018), thymus (Deguise et al., 2017), blood vessels (Somers et al., 2016), gastrointestinal tract (Sintusek et al., 2016; Wan et al., 2018), and muscle (Kim et al., 2020) have been reported. Sheng et al. (2018) reported that functional defects in the heart of severe Taiwanese SMA mice are cell-autonomous defects that are caused by Birc5-mediated cell cycle arrest and apoptosis. Several peripheral tissue lesions occur early in the development, even before motor neuron lesions, suggesting that they are a cause rather than a consequence of SMA. Recent studies have demonstrated that SMN are required throughout the lifespan of SMA mice, with peripheral tissues such as muscles being more demanding than motor neurons (Zhao X. et al., 2021). Although most studies have been conducted on mice and the reproducibility of their findings needs verification in patients, it is undeniable that these studies have expanded the scope of SMA from just a neurological disease to systemic disease. However, two questions remain unanswered. The first is how the low basal levels of SMN cause peripheral tissue lesions, like that discussed in section 5–1; a derived question is whether SMN has a unique downstream molecule or mechanism in neurons and peripheral tissue. The second question is how peripheral tissue affects the degeneration of motor neurons. A convincing hypothesis is that at least one of the peripheral tissues secretes neurotoxic substances due to the deficiency of SMN, resulting in the degeneration of motor neurons. Therefore, multi-tissue, multi-organ, and systemic studies are needed.

There is increasing evidence that the microbial community regulates neurological functions through the microbiota-brain-gut axis and this interaction is involved in the pathogenesis of several neurodegenerative diseases, such as Parkinson’s disease (PD), Alzheimer’s disease (AD), ALS, Multiple sclerosis (MS), and Huntington’s disease (HD; Quigley, 2017; Chandra et al., 2020). Wan et al. (2018) reported the disruption of the barrier function in the intestine of mice with severe SMA, along with a marked increase in the number of microbial communities in several peripheral tissues, such as the heart, liver, lung, spleen, and kidney. They have not identified the bacterial species and do not know if they affect the CNS. However, the microbiota-brain-gut axis remains one of the possible mechanisms through which peripheral tissues affect the CNS in the pathogenesis of SMA, although relevant studies are required.

### Potential of CRISPR technology in the treatment of SMA

The widespread application of the clustered regularly interspaced short palindromic repeats (CRISPR) technology, known for its powerful gene-editing capabilities, has yielded new strategies for the treatment of SMA. Different from several SMA treatment strategies described above, CRISPR acts as an “editor” rather than a “regulator”, with the T6C transition of the *SMN2* gene and several ISS and ESS being the editable targets. The mechanism of CRISPR and its applications have been described previously (Zhang, 2021). Zhou et al. (2018) successfully converted the *SMN2* gene to *SMN1* in the iPSCs of SMA patients using CRISPR/Cpf1 and a segment of single-stranded oligodeoxynucleotide, which facilitated the rescue of iPSCs-derived motor neurons. Using a CRISPR/Cas9 system with an adenine base editor, Wanjin Chen’s team at Fujian Medical University achieved the base replacement of two ESS sites on *SMN2* exon 7 in an SMA mouse model and successfully extended the lifespan of SMA mice (Lin et al., 2020). Another study by Chen’s team used the CRISPR/Cas9 system to delete the ISS element in the intron 7 of *SMN2*, and achieved correction of *SMN2* splicing and increased SMN protein in SMA patient cells. Importantly, the injection of their CRISPR/Cas9 system into zygotes increased the median lifespan of SMA mice by ~40-fold (10d vs. 400d; Li et al., 2019).

There are several major obstacles in the treatment of SMA through the editing of the *SMN2* gene using CRISPR technology. One is the low effectiveness of editing (<10%). Second, such gene editing is heritable and has ethical issues. Third, CRISPR technology has off-target effects and may also cause genomic instability, making it difficult to ensure its safety *in vivo* (Papathanasiou et al., 2021). Zhang Feng’s lab developed an RNA-editing system, CRISPR/Cas13, which could achieve RNA base-switching in mammalian cells (Abudayyeh et al., 2017; Cox et al., 2017). They subsequently optimized this technique to make it more conducive to viral packaging and *in vivo* delivery, making it more suitable for clinical treatment (Kannan et al., 2022). The CRISPR/Cas13 system can avoid ethical issues arising from DNA editing and is not only suitable for the treatment of SMA but also other genetic diseases.

## Conclusion and Outlook

The advent of nusinersen has saved several SMA patients and represents a breakthrough towards completely overcoming SMA. Its success is the result of the joint efforts of several scientists and doctors. The scientific discoveries and research ideas in the process of development of nusinersen have guided the development of other drugs. Despite the availability of drugs, there are still several fundamental scientific questions about SMA, which require detailed investigation. Continued investigations of such questions may uncover new therapeutic targets and lead to novel drugs. Moreover, the advancement of technology has introduced novel strategies for SMA treatment.

## Author Contributions

JQ, LW, RQ, and JS came up with ideas and participated in discussions. JQ, LW, RQ, TJ, JB, LS, PF, and JS prepared the manuscript. JQ and JS provided project administration and edited the revised manuscript. All authors contributed to the article and approved the submitted version.

## Funding

Funding support from National Science Foundation of China (No: 32000841) and Innovation and Entrepreneurship Program of Jiangsu Province (No: JSSCBS20211113) to JS, Innovation and Entrepreneurship Program of Jiangsu Province (No: JSSCBS20211605) to JQ.
